# Application of Genotyping-by-Sequencing on Semiconductor Sequencing Platforms: A Comparison of Genetic and Reference-Based Marker Ordering in Barley

**DOI:** 10.1371/journal.pone.0076925

**Published:** 2013-10-03

**Authors:** Martin Mascher, Shuangye Wu, Paul St. Amand, Nils Stein, Jesse Poland

**Affiliations:** 1 Leibniz Institute of Plant Genetics and Crop Plant Research, Gatersleben, Germany; 2 Department of Agronomy, Kansas State University, Manhattan, Kansas, United States of America; 3 United States Department of Agriculture, Agricultural Research Service, Hard Winter Wheat Genetics Research Unit, Manhattan, Kansas, United States of America; Universidad Miguel Hernández de Elche, Spain

## Abstract

The rapid development of next-generation sequencing platforms has enabled the use of sequencing for routine genotyping across a range of genetics studies and breeding applications. Genotyping-by-sequencing (GBS), a low-cost, reduced representation sequencing method, is becoming a common approach for whole-genome marker profiling in many species. With quickly developing sequencing technologies, adapting current GBS methodologies to new platforms will leverage these advancements for future studies. To test new semiconductor sequencing platforms for GBS, we genotyped a barley recombinant inbred line (RIL) population. Based on a previous GBS approach, we designed bar code and adapter sets for the Ion Torrent platforms. Four sets of 24-plex libraries were constructed consisting of 94 RILs and the two parents and sequenced on two Ion platforms. In parallel, a 96-plex library of the same RILs was sequenced on the Illumina HiSeq 2000. We applied two different computational pipelines to analyze sequencing data; the reference-independent TASSEL pipeline and a reference-based pipeline using SAMtools. Sequence contigs positioned on the integrated physical and genetic map were used for read mapping and variant calling. We found high agreement in genotype calls between the different platforms and high concordance between genetic and reference-based marker order. There was, however, paucity in the number of SNP that were jointly discovered by the different pipelines indicating a strong effect of alignment and filtering parameters on SNP discovery. We show the utility of the current barley genome assembly as a framework for developing very low-cost genetic maps, facilitating high resolution genetic mapping and negating the need for developing *de novo* genetic maps for future studies in barley. Through demonstration of GBS on semiconductor sequencing platforms, we conclude that the GBS approach is amenable to a range of platforms and can easily be modified as new sequencing technologies, analysis tools and genomic resources develop.

## Introduction

Rapid advancements in DNA sequencing technologies and platforms are lowering sequencing cost while increasing output. This is bringing routine sequencing of whole genomes within reach [1-6]. For larger and more complex genomes, sequencing approaches targeting a reduced representation of the full genome space are needed to reproducibly sequence the same regions across many samples [7]. Furthermore, for many genetic studies, a limited number of markers is sufficient and per sample cost considerations are important for assaying large populations. In many plant genetics studies and plant breeding applications large populations are needed for dissecting quantitative traits and selecting improved varieties [8-11]. In addition, diversity studies and phylogenetic analysis benefit from surveying a large number of individuals or accessions in natural populations or germplasm collections. Efforts are currently underway to genotype and/or sequence entire gene-bank collections for important crop species [12]. Robust and low-cost approaches for whole-genome characterization are needed to assay these large populations and collections.

Genotyping-by-sequencing (GBS) has been developed as a low-cost approach for reduced representation sequencing [13,14] and been demonstrated as a robust method for genome-wide profiling of complex populations [15]. GBS uses restriction enzymes to reproducibly capture a targeted portion of the genome enabling high levels of multiplexing while obtaining sufficient sequencing coverage. GBS has been successfully applied for a range of studies including genetic mapping [13,14,16,17], assaying genetic diversity and population structure [15], and genomic selection [18]. GBS is one variation of a range of approaches in the area of multiplexed sequencing of reduced representation libraries [13,14,16,19-25]. While a number of different restriction enzymes and adapter combinations have been employed, the commonality of these approaches is the use of restriction enzymes to capture a reduced representation of the genome. This targeted portion of the genome flanking restriction sites is ligated to DNA-barcoded adapters that enable multiplexed sequencing of many individuals on a single sequencing run. To date, the use of GBS approaches has largely focused on sequencing with the Illumina GAII and HiSeq platforms [25]. Recently, the use of semiconductor devices for non-optical genome sequencing has been demonstrated [26] and the technology has currently become available for routine sequencing with the Ion Torrent PGM and Proton (Life Technologies, Carlsbad, CA).

Illumina and Ion Torrent both utilize massively parallel sequencing-by-synthesis but use different chemistry and detection devices. The Illumina chemistry uses fluorescently labeled deoxynucleotide triphosphates (dNTPs) and employs optical sensors to detect the incorporation of the different nucleotides. The nucleotide labels serve as terminators for polymerization so that only a single nucleotide is incorporated during each cycle. The incorporated nucleotide is determined by imaging followed by cleavage of the label for the next cycle. In contrast, the Ion Torrent sequencing chemistry uses native dNTPs and electronic sensors to detect the release of hydrogen ions as dNTPs are incorporated into a growing DNA strand. To distinguish between different nucleotides, microwells containing the template strand and a polymerase are sequentially flooded with each dNTP. To detect the length of homopolymer runs, the sensor must detect the magnitude of the pH change to determine how many nucleotides were incorporated. Thus, errors on the Ion Torrent platform are mostly insertions and deletions in homopolymer runs, resulting from difficulties in evaluating the magnitude of signal when several dNTPs are incorporated in one cycle [27]. Since only a single base is incorporated each cycle, the Illumina chemistry may give more accurate sequencing through repetitive sequences and homopolymers.

As sequencing technologies develop, adapting current GBS approaches to new platforms will leverage the full advancements in sequencing output. The Illumina and Ion Torrent platforms differ in aspects such as the sequencing primers, the indexing method for sequencing multiplexed libraries and the read layout. The HiSeq2000 is capable of paired-end reads (i.e. sequencing the same fragment from both ends) and reading an incorporated bar code with an index read. With Illumina chemistry, all raw reads are of the same length, while the Ion Torrent performs single-end sequencing and outputs reads of variable length.

Understanding the application of GBS across different sequencing platforms will help to adapt this approach for future technology developments. Keeping pace with the technical advances in DNA sequencing, novel bioinformatic algorithms and pipelines for the analysis of sequencing are also being developed. Commonly used software programs to analyze GBS data include the dedicated pipelines in TASSEL (reference-based and *de novo* pipeline, UNEAK) [13,15,28] as well as pipelines that integrate several independent stand-alone tools [19,29,30], such as BWA [31], SAMtools [32] and CD-HIT [33].

With over 5 Gb of DNA, Barley (*Hordeum vulgare* L.) has one of the larger genomes among agronomically significant species [34]. As such, it is a good specimen with a large and complex genome, necessitating complexity reduction for sequence-based genotyping. Despite its size and complexity, a sequence-enriched physical and genetic map of barley was recently completed and serves as an excellent resource for future genetic studies and crop improvement in this important cereal crop [35,36]. Barley is a diploid and can serve as good model for grasses and specifically, bread wheat (*Triticum aestivum* L.), which has an even larger and more complex, polyploid genome of 17 Gb [34].

## Materials and Methods

### Plant Material

We genotyped a set of 94 recombinant inbred lines (RILs) from a cross between barley cultivars ‘Morex’ and ‘Barke’ that were previously used for high-throughput SNP genotyping to genetically anchor the physical map of barley [36,37]. The RILs were F_8_ derived lines from single seed descent from independent F_2_s. The population is available upon request to Nils Stein. DNA was extracted from two weeks old greenhouse grown seedlings using the protocol of Stein et al. [38].

### Adapter Design

**Figure 1:**
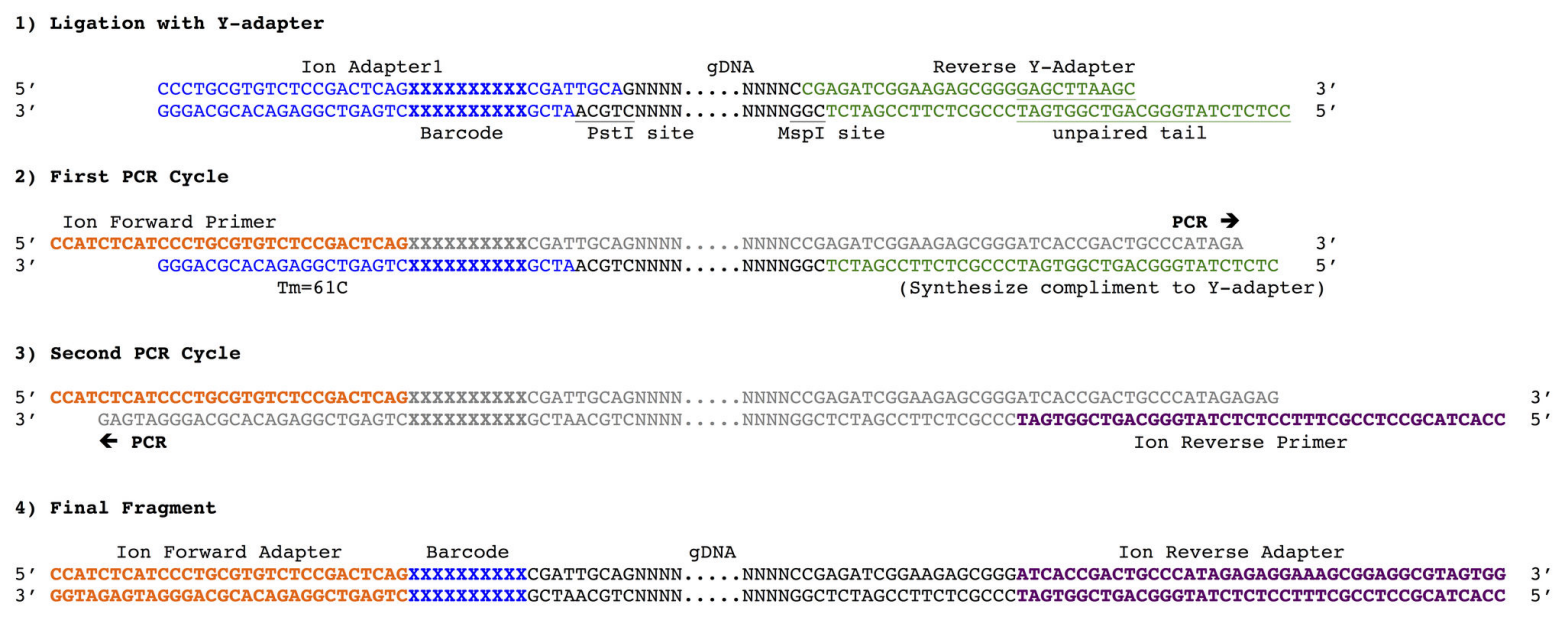
Design of genotyping-by-sequencing adapters for use with Ion Torrent sequencing chemistry. 1) Genomic DNA (black) is digested with a combination of *Pst*I and *Msp*I producing fragments with corresponding 3’ TGCA (*Pst*I) and 5’ CG (*Msp*I) overhangs. The barcoded forward Ion Adapter 1 (blue) is ligated to the *Pst*I generated overhang and the common Ion Adapter 2 (green) is ligated to the *Msp*I generated overhang. The variable bar code is in bold and the unpaired tail of the Y-adapter is underlined. 2) During the first PCR cycle, the forward primer (orange) binds to the corresponding Adapter 1 site and proceeds to synthesize the complementary strand (grey) to the genomic sequence tag and then the unpaired tail of the Y-adapter. The common *Msp*I-*Msp*I fragments (not shown) have a Y-adapter on both ends and lack a complementary binding site to initialize PCR amplification. 3) During the second and subsequent rounds of PCR, the reverse primer (purple) can bind to the newly synthesized complement and initialize synthesis on the reverse strand. These PCR reactions continue until completion of the fully synthesized fragments. 4) The final fragment is ready to sequence and consists of the Ion Torrent forward priming site (orange) with a bar code (blue) followed by the genomic sequence fragment (black) and the Ion Torrent reverse priming site (purple).

We designed a set of adapters for the two-enzyme GBS approach [14] that were matched to the primers of Ion Torrent sequencing platforms (Figure 1). We used the same enzyme combination of *Pst*I and *Msp*I as in a previous study [14]. The forward adapter was designed with 21 bp of the Ion Forward adapter followed by a 9 bp bar code. An additional 4 bp ‘CGAT’ sequence was added following the bar code to increase annealing stability for the double-stranded adapters. Adapter 1 has a 4 bp 3’ overhang of ‘TGCA’ corresponding to a *Pst*I restriction site. In contrast to the Illumina sequencing chemistry, the Ion platform does not necessitate a balanced set of bases. Previous bar codes for GBS on the Illumina platform were designed of varying lengths to stagger the restriction cut-site and avoid a single nucleotide representation at a given position in the first 12 bp of the sequencing run [13,14]. This balanced base composition is not needed with the Ion chemistry, therefore all bar codes were 9 bp in length. For initial testing, a set of 24 bar codes were used to make a 24-plex sequencing library (Table S1). For further studies, sets of 96 and 384 bar code adapters were designed (Table S2).

With a two-enzyme GBS approach, a rare-cutting and a frequent-cutting enzyme are used for co-digestion to produce fragments that are uniformly Adapter 1 and Adapter 2 on each respective end of the fragment [13,14]. For sequencing with the Ion chemistry, we modified previous adapter designs to develop a Y-adapter with the Ion reverse priming site. The priming site is used as the 5’ unpaired tail of the Y-adapter to prevent amplification of the more common *Msp*I-*Msp*I fragments. During PCR amplification, the complement of the unpaired tail on the Y-adapter is synthesized from the opposite end of the fragment containing the *Pst*I restriction site and the corresponding forward adapter. With this approach, only the *Pst*I-*Msp*I fragments will amplify as the more common *Msp*I-*Msp*I fragments have a Y-adapter on both ends and PCR amplification is not initialized [14].

The PCR primers were designed as the full Ion sequencing primer sites. During the first round of PCR the forward primer will bind to and extend the forward adapter. Synthesis from this priming to the other end of the fragment will create the complementary strand of the Y-adapter. The reverse primer now has a binding site and will extend to the full reverse sequencing primer site during the second round of PCR. The fully synthesized fragment will contain a 30 bp forward adapter followed by a 9 bp bar code. At the end of the genomic DNA the 60 bp common adapter contains the 41 bp reverse priming site for Ion sequencing (Figure 1).

### Library Construction and Sequencing

A full, detailed protocol of the adapter/primer design, library construction and sequencing conditions is included as supplemental material (Text S1, Table S3). We constructed four 24-plex libraries for sequencing on the Ion Torrent PGM and the Ion Torrent Proton platforms. Following the approach of Poland et al. [14], genomic DNA was quantified using PicoGreen (Invitrogen, Carlsbad, CA) and normalized to 20µL of 10ng/µL (200ng total) in 96 well plates. Restriction digest buffer (NEB4) and restriction enzymes *Pst*I and *Msp*I (New England Biolabs, Ipswich, MA) were added to each sample. Samples were incubated at 37°C for 2 hours for a complete digestion followed by 65°C for 20 minutes to inactivate the enzymes. Ligation of the adapters was completed in the same plate. First, 0.1 pmol of Adapter 1 (unique for each sample) and 15 pmol of Adapter 2 (common adapter) were added to each well. A ligation master mix consisting of NEB Buffer 4, ATP, and T4 ligase, was then added to each well. Ligation reactions were completed at 22°C for 2 hours followed by 65°C for 20 minutes to inactivate the enzymes. Following adapter ligation, samples were pooled in 24-plex and cleaned-up using QIAquick PCR purification kits (Qiagen, Valencia, CA). The libraries were then PCR amplified. For each library four independent 25 µL PCR reactions were completed and then pooled. Each 25 µL reaction was performed with 5 µL DNA template (the prepared library), 5 µL of NEB 5x master mix, 2 µL of Ion PCR forward and reverse primers at 10 µM each, and 13 µL H_2_0.

Two libraries were first sequenced on the Ion Torrent PGM™ Sequencer using Ion 318™ sequencing chips. The first library was sequenced on a total of 6 chips and the second library on 4 chips. Libraries were quantified using the 2100 Bioanalyzer High Sensitivity DNA kit (Agilent, Santa Clara, USA). Templated spheres were generated with the Ion OneTouch™ 200 Template kit using 35 million library molecules and controlled with the Ion Sphere™ Quality Control Kit before being loaded on an Ion 318 Chip for sequencing using the Ion PGM™ 200 Sequencing kit (Life Technologies, Carlsbad, USA).

The last two libraries were sequenced on the Ion Torrent Proton™ System using Ion PI™ Chips with each library sequenced on two chips. Libraries were sized using the Experion system (BioRad, Hercules, CA) and normalized to a working concentration of 10 pM. Libraries were then diluted to 1 pM and Ion sphere particle emulsion PCR amplification was completed on an Ion OneTouch™ 2 instrument according to the recommendations in the Ion PI Template OT2 200 Kit (Life Technologies, Carlsbad, CA). After emulsion PCR, a Qubit Ion sphere quality control assay was performed to verify that percentage of templated ion sphere particles (ISPs) was in the optimal range of 10% to 25% prior to enrichment and sequencing. The loaded chips were sequenced with the Ion PI™ Sequencing 200 kit.

Using the protocol from Poland et al. [14], a 96-plex library was made from the same samples for Illumina sequencing using the 384A adapter set. This library was sequenced on a single lane of Illumina HiSeq 2000 (Illumina, San Diego, CA) using a single end 100 bp run.

Sequence reads for all Ion Torrent and Illumina runs have been deposited in the NCBI SRA archive SRP010876 with experiment number SRX329196 under BioProject PRJN214367 and are also available at www.wheatgenetics.org.

### Sequence Data Analysis and Development of Reference-based Marker Maps

Sequence reads from Fastq files were demultiplexed with a custom C program (Text S2). Adapter trimming was performed with cutadapt (http://code.google.com/p/cutadapt). Reads shorter than 30 bp after adapter removal were discarded. Trimmed reads were mapped against the whole-genome shotgun assembly of barley cultivar Morex [36] with BWA version 0.6.2 [31] (commands aln and samse). The BWA command aln was called with the parameter “-q 15” for quality trimming, otherwise default parameters were used. SNP calling was performed with the SAMtools / BCFtools pipeline version 0.1.18 with default parameters [32,39]. The additional parameter “-D” was used for samtools mpileup to obtain per sample read depth. Genotype calls were filtered with a custom AWK script (Text S3) selecting only SNPs matching the following criteria: (i) read depth at least 1 (3) and genotype quality at least 3 (5) for homozygous (heterozygous) calls, (ii) at most 90% missing genotype calls (genotype calls with insufficient coverage or quality were set to missing), and (iii) minor allele frequency at least 5% (iv) less than 10% heterozygous calls. These parameters were chosen specifically for a RIL population. For RILs, we expected a residual heterozygosity of 1-2%. We expect a comparable rate of heterozygous calls erroneously called homozygous when only one allele was sampled. We accepted this error rate rather than using a higher stringency under which criteria much fewer loci could be genotyped. For an unselected biparental population, we expect a 50% minor allele frequency. However, to account for missing data and stochastic sampling, the minimum minor allele frequency was set to 5%.

The TASSEL GBS pipeline was used to call SNPs in tags prior to alignment on the reference genome [13,28]. Using the *de novo* GBS pipeline, 64bp tags from TASSEL were mapped against the Morex whole-genome shotgun assembly [36] with BWA aln/samse [31] with default parameters. Only mapped tags with quality score >= 30 were retained. A custom R script was used to convert SNP positions on tags to positions on Morex WGS contigs by evaluating the CIGAR string in the BWA output. Genetic and physical locations of the barley physical framework [36] were used to position SNPs from the SAMtools pipeline or mapped tags from the TASSEL pipeline. Marker positions and associated genotypes calls from different datasets were compared with custom R scripts. SNPs were merged based on their coordinates on the Morex WGS assembly. Only SNPs with less than 50% missing data and minor allele frequency >= 30% were considered for the comparison. The intersection between SNP sets was visualized using the R package “vennDiagram” [40].

To construct a *de novo* genetic map, we utilized the MSTmap software [41]. Prior to map development, the GBS markers for the PGM and Proton datasets were merged based on the Morex WGS assembly. Markers with more than 20% missing data were discarded. A single marker was selected per whole-genome shotgun sequence assembly contig when multiple tags aligned to the same contig. In total, 1,817 markers were used as input for MSTmap. The parameters of MSTmap were set to: population type = DH, distance_function = kosambi, no_map_dist = 20, no_map_size = 2, missing_threshold = 0.1, cut_off_p_value = 1e-05. The population type was set as ‘DH’ (doubled haploid) as recommended by the manual for advanced RIL populations. The missing threshold removes all markers with more than 10% missing or heterozygous calls. The cut off p-value specifies a threshold for clustering markers into linkage groups and was set to 1E-05. The no_map parameters are used to discard small sets of isolated markers. As all markers went into one of seven linkage groups, this parameter was of no consequence.

All markers were assigned to one of seven linkage groups, which corresponded to the seven barley chromosomes. Most of the markers (1,584 out of 1,817; 87.2%) were located on contigs anchored to the integrated physical and genetic map of barley. Spearman’s rank correlation coefficient between marker order of the *de novo* genetic map and the reference (International Barley Sequencing Consortium; IBSC) map was calculated per chromosome. Marker positions were compared by performing loess regression (R functions loess and predict) between cM positions in the *de novo* map and the IBSC map.

## Results and Discussion

### Genotyping-by-sequencing on semiconductor platforms

**Figure 2:**
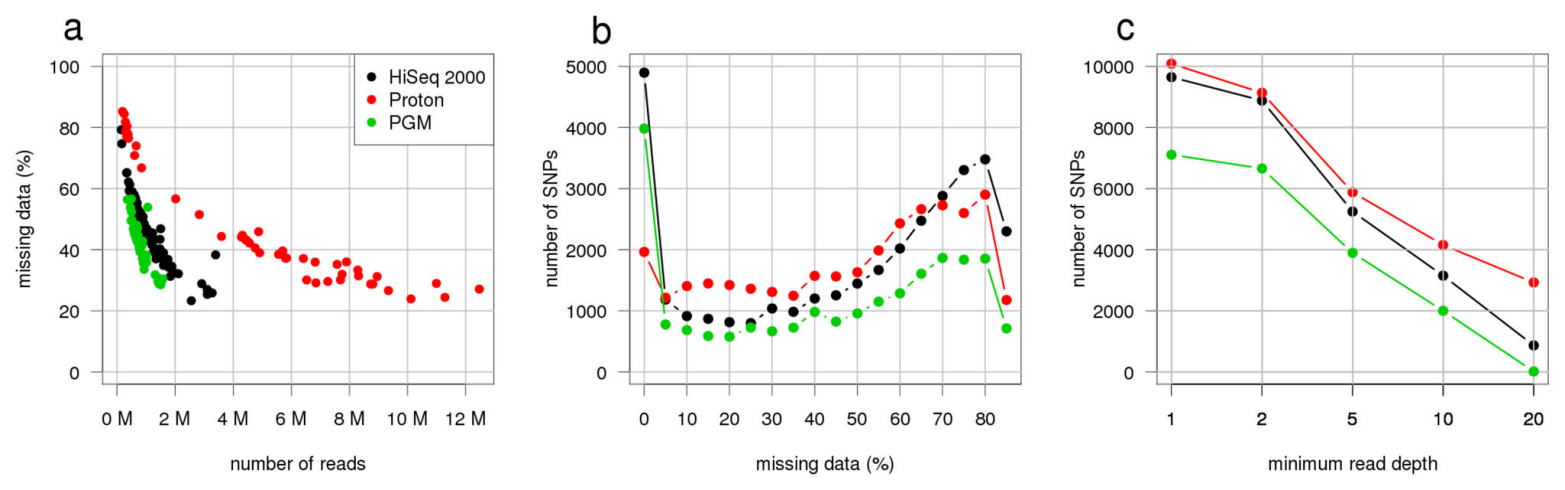
Relationship between number of sequence reads, missing data, sequencing depth and the number of SNP calls. (**a**) The average percentage of missing data per SNP in each sequenced sample is plotted as function of the number of sequence reads in that sample. (**b**) Histogram of missing data per SNP. (**c**) The number of SNP calls plotted against the minimum depth at a variant position in a given sample to make a successful genotype call. All SNP calls were made with the SAMtools pipeline. The minor allele frequency was set to 30% and the maximum rate of missing data was set to 50%. The sequencing platforms used for this study include Illumina HiSeq2000 (black), Ion Torrent PGM (green) and Ion Torrent Proton (red). The color code for all panels is given in the legend to (**a**).

We developed a total of four libraries comprised of Morex and Barke barley cultivars and 94 unique RILs from these two parents for testing GBS on semiconductor sequencing platforms. Two of these libraries were sequenced with Ion Torrent PGM and two with Ion Torrent Proton. From six PGM 318 chips we obtained a total of 30M reads for the first library and 17M for the second library. On the Proton we ran two PI chips for each library obtaining 52M, 39M, 73M, 85M, sequence reads for each of the chips respectively. The pooling for the library sequenced of the PGM (Figure 2a) was more uniform (350,000 to 1.6 M reads per samples), when compared to the Proton, where reads count per sample ranged between less than 1 M and to over 12 M reads. From one lane of HiSeq2000, we obtained 128M sequence reads on a 96-plex GBS library of the same RILs.

At the first filtering step sequence reads were checked for an exact match to one of the bar codes followed by the expected *Pst*I restriction site. We found that the number of usable reads for each platform was in the range of 90-95%. For example, each of the Proton runs yielded ~92% of the sequence reads with a good bar code followed by a *Pst*I restriction site. This is an average range for sequenced GBS libraries, indicating a robust construction of the library with the *Pst*I target sequences.

For sequencing on the Ion Proton™ System, we found several modifications of the standard sequencing protocol improved bead loading and sequencing results. The Life Technologies Ion PI Template OT2 200 Kit recommends that stock sample libraries be diluted to a concentration of ~26 pM for use in the ion sphere particle emulsion PCR. Based on our quantification, we found that PCR amplified sample libraries need to be diluted to ~1 pM concentration in order to produce the percentage of templated ion sphere particles (ISPs) in the optimum 10% to 25% range. For example, after emulsion PCR, sample libraries in the 8 pM concentration range often produced an excess of 80% templated ISPs, which would likely produce nearly 100% polyclonal ISPs and very poor sequencing results. PCR amplified GBS libraries are very concentrated and will often need to be diluted in excess of 100,000X. Care should be taken to dilute the sample in a series of steps so that each dilution is never greater than 100X and that a minimum of 2 µL of stock is used for each dilution. Diluted libraries were used immediately without a storage period.

### Reference-based Marker Ordering

**Figure 3:**
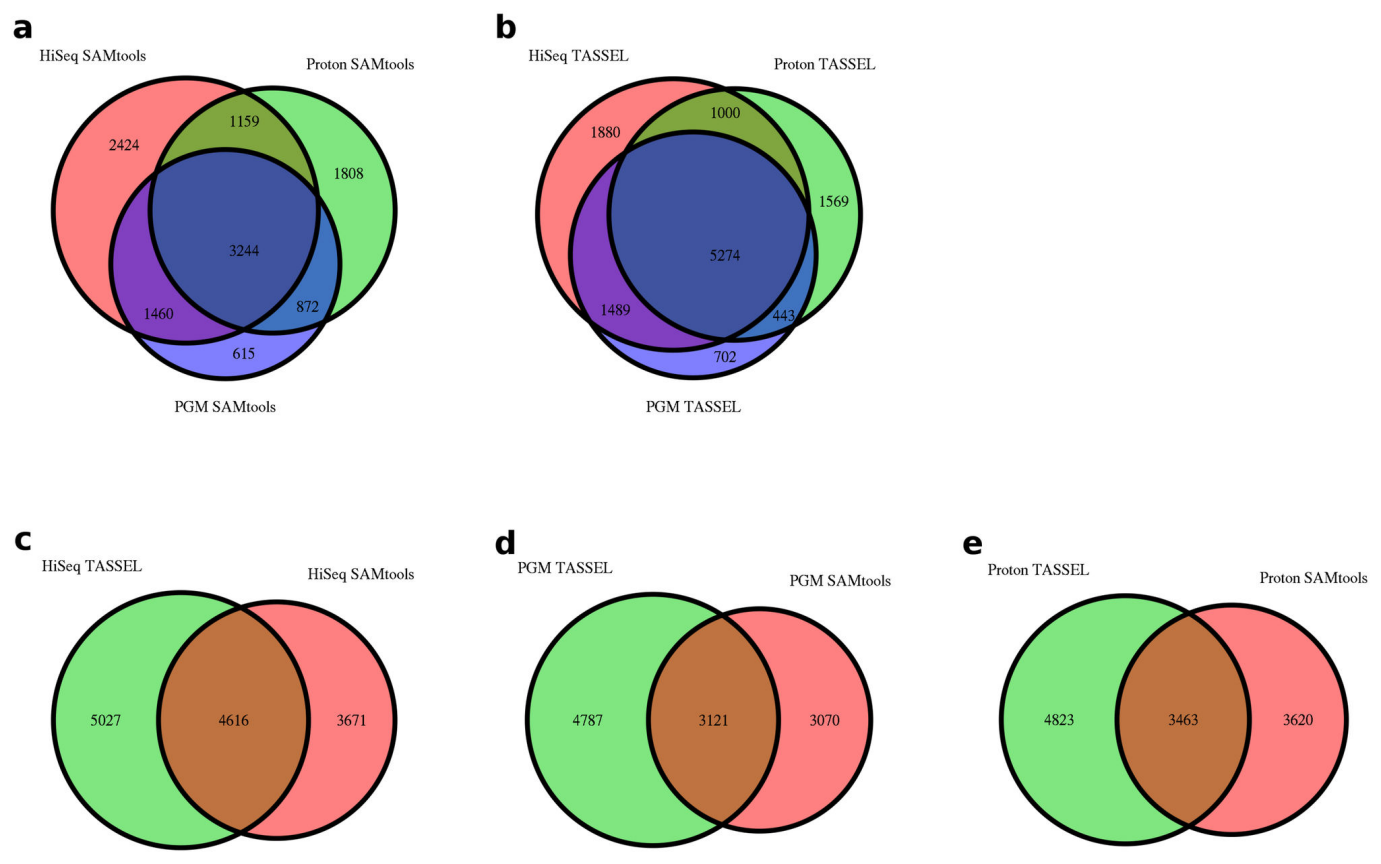
Venn diagrams of the number of SNPs identified in each dataset and with the respective bioinformatics pipeline. (**a**) SNPs identified with the SAMtools pipeline across all three platforms. (**b**) SNPs identified with the TASSEL pipeline across all three platforms. (**c**) SNPs identified in the HiSeq2000 data with both pipelines. (**d**) SNPs identified in the PGM data with both pipelines. (**e**) SNPs identified in the Proton data with both pipelines.

We evaluated two different SNP calling and mapping pipelines in the barley datasets; TASSEL and SAMtools (Dataset S1). The TASSEL pipeline identified fewer SNPs that could be anchored to the reference assembly but more of these SNPs remained after filtering for minor allele frequency (MAF) and proportion present (Table 1). For each of the pipelines and datasets 7,000 to 10,000 SNPs were identified at MAF > 0.3 with data present for a majority of the lines. The number of SNP calls increased to over 30,000 if SNPs with up to 90% missing data were tolerated. Using different coverage thresholds (1x to 20x) reduced the number of SNPs, but even with 10x coverage all platforms provided at least 1,000 SNPs with less than 50% missing data (Figure 2c). There was a paucity of SNPs that were identified in common between the two pipelines (Figure 3), suggesting that the different alignment and filtering criteria between the pipelines played a significant role in which SNPs were retained in the final data sets. The TASSEL pipeline only considered 64 bp tags (including the invariable 4 bp TGCA cut-site) while the SAMtools pipeline also calls SNPs at the ends of 100 bp reads. For example, 1,726 SNPs were detected by the SAMtools pipeline that were more than 60 bp away from the *Pst*I restriction site. Similarly, 367 SNPs found by SAMtools in the Proton data were farther than 100 bp away from the restriction site. These SNPs were not reached by the 100bp HiSeq run. In contrast, the TASSEL pipeline is likely more robust to SNP discovery, particularly in duplicated sequences, as it uses a population-based filtering to identify properly segregating SNPs [18].

**Table 1:** The number of SNPs positioned on the Morex reference assembly for Morex x Barke recombinant inbred lines using genotyping-by-sequencing on three different sequencing platforms; HiSeq 2000, Ion Torrent PGM and Ion Torrent Proton.

**Platform dataset (bioinformatics pipeline)**	**HiSeq 2000 (SAMtools)**	**HiSeq 2000 (TASSEL)**	**Ion PGM (SAMtools)**	**Ion PGM (TASSEL)**	**Ion Proton (SAMtools)**	**Ion Proton (TASSEL)**
Number of raw reads	257 M	257 M	47 M	47 M	249 M	249 M
Number of SNPs	33,634	19,242	21,805	13,640	32,621	17,659
Number positioned on Morex assembly	33,634	14,853	21,805	10,610	32,621	11,697
Percent positioned on Morex assembly	100.0%	77.2%	100.0%	77.8%	100.0%	66.2%
Number positioned (MAF >= 0.3 & present >= 0.5)	8,287	9,732	7,055	9,025	7,083	11,091
Number positioned with unique positions (MAF >= 0.3 & present >= 0.5)	8,287	9,643	7,055	7,908	7,083	8,286

Two different bioinformatics pipelines, SAMtools and TASSEL, that were used for SNP identification and genotype calling are shown in parenthesis under the platform.

The independent construction and sequencing of libraries for the Ion and Illumina platforms likely contributed to the different SNPs identified between the pipelines simply due to the probability of sampling the same sites with low coverage on the libraries. For example, the libraries sequenced on the Ion Torrent Proton had a very uneven read distribution (Figure 2a) resulting in a smaller number of SNPs with less than 5% missing data (Figure 2b). In contrast, the library sequenced on the Ion Torrent PGM was well balanced and the resulting number of high-quality SNP (< 50% missing data) was better than would have been expected from the five-fold lower amount of sequence reads when compared to either HiSeq2000 or Proton. This highlights that library uniformity may be an even more crucial determinant of final data quality than the overall amount of sequencing data.

It was clear that the different SNP calling pipelines were primarily responsible for differences in the SNPs identified. For example, using the same HiSeq dataset, the TASSEL and SAMtools pipelines identified 4,616 in common but sets of 5,027 and 3,671 were unique to the TASSEL and SAMtools pipelines, respectively (Figure 3). Although differences were observed between the pipelines and platforms on which SNPs were discovered, there was high concordance on genotype calls for common SNPs. There was strong agreement in positioning of SNPs from the different pipelines against the reference, for assigning parent of origin to RILs, and for individual SNP genotype calls. When using the same bioinformatics pipeline, there was only 1-2% discordance between the different platforms for genotype calls on shared SNPs (Table 2). For example, the Ion Proton and HiSeq datasets had a 99% agreement in genotype calls when analyzed with the SAMtools pipeline. Using two different bioinformatics pipelines introduced a rate of about 1% discordant calls. With the same HiSeq dataset, the SAMtools and HiSeq pipelines had 0.77% discordant genotype calls. Overall the Ion PGM Sequencer had the highest rate of discordant calls at around 2% when calls made with the same pipeline were compared across platforms. Given a 1-2% rate of discordant calls between platforms, it is expected that the actual error rate for a given genotype is less than 1% on any one of the platforms. For example, a 0.5% error rate on each side of two imperfect genotyping platforms will sum to 1% discordant genotypes when comparing the two sets. This level of discordant genotype calls can also be attributed to low coverage of heterozygous regions, which are expected at 1-2% of the genome for these RILs.

**Table 2:** Comparison of single nucleotide polymorphism (SNP) genotype calls for Morex x Barke recombinant inbred lines from genotyping-by-sequencing datasets using three different sequencing platforms (Illumina HiSeq 2000, Ion Torrent Proton and Ion Torrent PGM).

**Set 1**	**Set 2**	**shared SNPs**	**shared samples**	**% discordant genotype calls**
HiSeq (SAMtools)	PGM (SAMtools)	4,704	48	1.51
HiSeq (SAMtools)	Proton (SAMtools)	4,403	48	1.02
HiSeq (SAMtools)	HiSeq (TASSEL)	4,557	96	0.77
HiSeq (SAMtools)	PGM (TASSEL)	3,556	48	1.93
HiSeq (SAMtools)	Proton (TASSEL)	2,451	48	1.28
HiSeq (TASSEL)	PGM (SAMtools)	3,007	48	2.38
HiSeq (TASSEL)	Proton (SAMtools)	3,299	48	1.58
HiSeq (TASSEL)	PGM (TASSEL)	5,756	48	2.35
HiSeq (TASSEL)	Proton (TASSEL)	4,432	48	1.48
PGM (SAMtools)	PGM (TASSEL)	2,494	48	0.95
Proton (SAMtools)	Proton (TASSEL)	2,197	48	0.76

Shown in brackets are two different bioinformatics pipelines used for identifying and calling SNPs in the population. Two different sets of samples were sequenced on the Proton and the PGM, so genotypes cannot be compared. For comparison involving TASSEL calls, only SNPs represented by unique tags were used.

**Figure 4:**
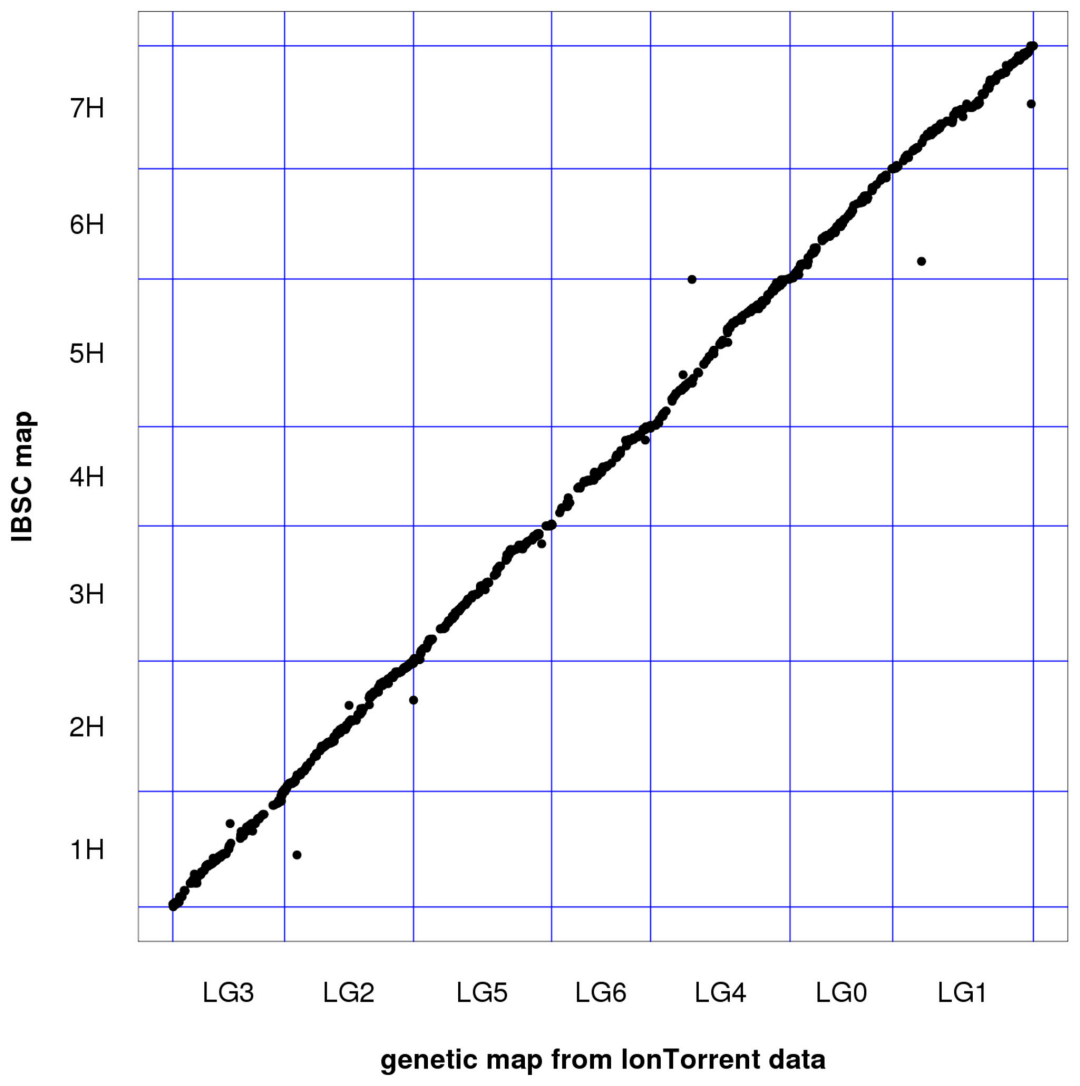
Genotyping-by-sequencing marker order based on the International Barley Sequencing Consortium reference framework (y-axis) compared to a *de novo* genetic order (x-axis) from 94 Morex x Barke recombinant inbred lines genotyped on either the Ion Torrent PGM or Proton platform. Each dot corresponds to one of 1,584 markers from the *de* *novo* map that was positioned to the physical and genetic framework of barley.

To assess the accuracy of the reference-based framework for constructing genetic maps, we compared an independently constructed genetic linkage map of 94 RILs with data from the PGM and Proton to the reference assembly. Overall there was high agreement on the linear order of GBS markers (Spearman’s rank correlation coefficient = 0.99) between the *de novo* genetic and reference maps (Figure 4). Each linkage group corresponded to one chromosome and only 2 out of 1,584 (0.1%) markers had discordant chromosome assignments. The marker order was highly consistent. Only an additional 62 markers (3.9%) were positioned farther than 5 cM away from each other in the *de novo* and the reference map. Overall, there was high agreement in the position and order of markers using a *de novo* genetic map compared to the reference framework.

## Conclusions

We developed a GBS protocol suitable for sequencing on the Life Technologies semiconductor sequencing platforms, Ion Torrent PGM and Ion Torrent Proton. No major changes to the GBS approach were needed, only modifications to the adapter sequences to enable annealing and sequencing with the Ion Torrent chemistry. We used the previous two-enzyme GBS approach employing a rare- and a frequent-cutting restriction enzyme combination *Pst*I and *Msp*I. This approach largely produces fragments that have the correct Adapter 1 (forward with bar code) and Adapter 2 (reverse) sequences on each respective end of the fragment [14]. In contrast, the single enzyme GBS approach produces a mixture of fragments with roughly half of the fragments expected to have the same adapter on each end [13]. These ‘uni-adapter’ fragments do not bridge amplify or sequence with the HiSeq chemistry and optimal loading of sequencing reactions can be accomplished by loading the flow-cell at a higher concentration. With the Ion chemistry, it is unclear if the same approach would be viable.

There was low error among the different sequencing platforms when comparing genotyping calls across inbred lines. We observed 1-2% discordance between Ion Torrent PGM, Ion Torrent Proton and HiSeq2000 platforms when using the same SNP calling pipeline. Given a true sequencing error rate (and matching genotyping error rate) of around 0.5-1%, this level of discordance between any two sequencing platforms would be expected. Due to almost complete homozygosity of RILs, we were able to accurately call genotypes at very shallow coverage (1x). For a population of heterozygous individuals (e.g. outbred species or F_2_s in selfing organisms), more stringent criteria have to be used. However, even at 10x coverage, we could still call several thousand SNPs.

There was high agreement between a *de novo* genetic map and the IBSC reference assembly. This supports the approach of using a reference-based framework for genetic maps in new barley populations. Both reference-based and *de novo* genetic maps, measure the distance between markers in terms of estimated recombination frequencies. However, the reference-based methods take marker order and distance not from the analyzed GBS data set, but use a pre-established marker order. In the case of barley, genetic markers from several populations typed with conventional assays, array-based genotyping as well as genotyping-by-sequencing were integrated to a framework map constructed by typing ~3000 markers in a population of 360 RILs [36,37]. The marker data were integrated with the physical map and survey sequence data (BAC end sequences and whole-genome shotgun contigs). This resource makes it possible to establish marker order in populations where it would be difficult or impossible to construct a robust genome-wide genetic-map such as in very small populations (less than 40 individuals), a set of selected individuals, or in advanced backcross populations. In addition, the physical and genetic map and associated whole-genome shotgun assemblies provides valuable information about gene content in the neighborhood of a given marker.

The great advantage of this reference-based approach to large bi-parental mapping populations is that higher levels of multiplexing can be employed. For GBS, higher multiplexing reduces the genotyping cost but gives lower sequence coverage for each sample and more missing genotype calls. The ordering of markers on a reference framework, however, reduces the need for complete data across the full population (i.e. more missing data can be tolerated), while there will still be a surplus of markers for identifying recombination breakpoints for any given line in the mapping population. At current sequencing costs with 192-plex GBS libraries, the per sample genotyping cost for new populations has dropped below $10 per sample while still producing very high-resolution genetic maps for mapping segregating traits. With Illumina HiSeq or Ion Torrent Proton, these levels of multiplexing are feasible and will still provide thousands of usable markers. Most “bench-top” sequencers, including the PGM, do not produce enough data on a single run to be useful for GBS. As in this study, multiple runs were needed to produce enough coverage on a 24-plex library, which will greatly increase the per sample cost for genotyping.

In this study we demonstrated the relative ease of transferring the GBS approach to new sequencing platforms. The strength of GBS for assaying large populations is dependent on obtaining as many independent sequence reads as possible [25]. This determines the amount of missing data as well as the level of multiplexing that can be reasonably utilized. As new sequencing platforms develop, GBS will be preferentially targeted to platforms that produce more reads rather than just longer reads [25]. This is in contrast to most applications on next-generation sequencing platforms, particularly those focused on whole-genome sequencing and assembly. In the era of massively parallel sequencing, development of platforms that are expected to produce billions of sequence reads per run is a promising prospect for future applications of GBS. As the number of sequenced genomes continues to grow, the application of reference-based ordering of GBS markers gives additional strength and utility to this low-cost genotyping approach.

## Supporting Information

Table S1 **Sequence of 24 bar code adapters designed for Ion Torrent as used in this study.**
(XLSX)

Table S2 **Sequence of 384 bar code adapters designed for Ion Torrent.**
(XLSX)

Table S3 **Sequencing runs and sample bar codes.**
(TXT)

Text S1 **Protocol for genotyping-by-sequencing library construction for Ion Torrent.**
(DOCX)

Text S2 **Custom C program for demultiplexing fastq files.**
(ZIP)

Text S3 **Custom AWK script for genotype calling.**
(ZIP)

Dataset S1 **Genotype calls for Morex x Barke RIL population.**
(ZIP)
