# Supplementary material for: Application of Genotyping-by-Sequencing on Semiconductor Sequencing Platforms: A Comparison of Genetic and Reference-Based Marker Ordering in Barley
Source: PLoS One. 2013 Oct 3;8(10):e76925. doi: 10.1371/journal.pone.0076925 (PMC3789676; doi:10.1371/journal.pone.0076925)
Supplement: Text S1 — Protocol for genotyping-by-sequencing library construction for Ion Torrent. (DOCX) [file pone.0076925.s004.docx]

**Library Creation for Genotyping-by-sequencing (*PstI*-*MspI*)**

**on Ion Torrent PGM and Proton**

J. Poland and S. Wu

USDA-ARS and Kansas State University

April 3, 2013

**ADAPTERS**

The adapters are ordered as normal oligos (IDT with standard desalting; 25uM amount). For each adapter two oligos must be ordered in complimentary pairs and must be annealed to form the double stranded adapter prior to use of the protocol. They are stable after annealing and can be stored at -20°C indefinitely. The adapters are best annealed by heating to 95°C and then slowly cooling to 30°C at a rate of -1°C/minute. This was accomplished a BioRad DNA engine by programming a single step PCR cycle at 95°C for 1 minute and then decreasing the temperature by 1°C each cycle for 65 cycles. The barcoded adapters can be ordered in a plate and all of the annealing completed in plate format. The adapters should be quantified after annealing to ensure that the double strand DNA formation was complete and they are at the correct concentration. Uniform concentration of adapters is critical to producing uniform numbers of reads between samples when sequencing the multiplexed library.

**QUANTIFY AND NORMALIZE DNA**

DNA concentration is very critical to producing even numbers of sequence tags from each sample. It is recommended that DNA be quantified using a florescence based quantification method such as PicoGreen.

**RESTRICTION DIGEST**

This protocol uses a double-digest with *Pst*I and *Msp*I. The *Pst*I overhang corresponds to Adatper1 (barcoded) and the second enzyme overhang corresponds to the common Adapter2 (y-adapter). The sequencing reaction proceeds from Adapter1 and sequences through the barcode. Paired-end reads are not used and the other end of the fragment is not sequenced.

NEB enzymes are used for the double-digest as they are optimized to work in the same buffer. *Pst*I and other common enzymes are used in NEB Buffer4.

**LIGATION**

The ligation is completed in the same tube/plate as the digestion. The ligation reaction is conducted in NEB Buffer4 with the addition of ATP (NEB T4 DNA Ligase #M0202). The concentration of Adapter1 needs to be adjusted depending on the species. For wheat and barley 0.05 to 0.1 pmol works well for 200ng of genomic DNA. The Adapter2 is a Y-adapter and can be added in excess, as it will not amplify unless the PCR reaction has first proceeded from Adapter1 on the other end of the same fragment. The ligase should be inactivated prior to multiplexing the samples by holding at 65°C for 20min after the ligation is complete.

NOTE: 50X more common Adapter2 than Adapter1. Adapter 2 is the Y-adapter so it can be added in excess.

**MULTIPLEXING**

The ligated samples are now multiplex and PCR amplified in a single tube. This produces a single library from 48 to 384 samples, which is sequenced on a single chip of Ion Torrent PGM or Proton. (Due to limited output, PGM is not recommended for species with large genomes or for higher levels of multiplexing)

**PCR AMPLIFICATION**

The multiplex library is now PCR amplified. This will select for fragments that have a *Pst*I cut-site and an *Msp*I (or second enzyme) cut-site. The *Msp*I- *Msp*I fragments will be common but will not amplify due to the Y-adapter. The *Pst*I - *Pst*I fragments will be very rare.

**Anneal Adapters**

1X Elution Buffer (EB) – 10mM Tris-Cl, pH 8.0-8.5

10X Adapter Buffer (AB) – 500mM NaCl, 100mM Tris-Cl

This is completed in plates for the barcoded adapters (Adapter1)

1. Suspend (dried) single-stranded adapter oligos to 100uM in 1X EB
2. Make 100ul of 10uM double stranded adapter:

10ul – each single stranded oligo (@ 100uM)

10ul – 10X AB

70ul – H_2_0

Heat to 95°C and cool at 1°C per/minute to 30°C. Hold at 4°C.

(program “anneal”)

1. Dilute adapters 3:10 (to ~3uM) and quantify using PicoGreen.
2. Normalize to 2.2 ng/ul (=0.1uM)

Make *Msp*I common reverse Y-adapter (Adapter2) - single tubes

*Common reverse adapters* – follow same steps but leave Adapter2 at 10uM for working stock.

**Working Adapter Stock**

Each well in the working adapter plate will have 0.02uM (unique) barcode Adapter1 and 3uM (common) Adapter2

In 96-well plate add:

20 ul – Barcoded Adapter1 @ 0.1uM

30 ul - MspI Adapter2 @ 10uM

50 ul – 1X AB

Mix well, spin down

**Normalize DNA**

1. Quantify DNA using PicoGreen. Protocol “PicoGreenDNAQuant.doc”

(DNA concentration must be between 20ng/ul and 150ng/ul for accurate pippetting. If too high, dilute before normalizing.)

**Restriction (20ul)**

10 ul – DNA (20ng/ul = 200ng total)

10ul – restriction master mix

*Pst*I-HF – NEB #R3140 (20,000 units/ml)

*Msp*I – NEB #R0106 (20,000 units/ml)

Restriction MasterMix:

Plate Sample

220 2.0 ul – 10X NEB Buffer 4

44 0.4ul – PstI-HF (5 units)

44 0.4ul – MspI (5 units)

792 7.2ul – H_2_0

QIAgility Program: “PstI-MspI_Normalize_Digest”

Normalize 10ul DNA @ 20ng/ul into 96-well plate.

Add 10ul Restriction MasterMix to DNA and mix.

Ligation: 37°C for 2 hour; 65°C for 20 min; hold at 8°C (program “reslig”)

NOTE: Proceed directly to ligation.

**Ligation (40ul)**

20 ul - restriction digest

5 ul - Adapters (0.02uM Adapter1 = 0.1pmol, 3uM Adapter2 = 15pmol)

15 ul - Ligation Master Mix

Ligation MasterMix:

Plate Sample

220 2.0 ul – NEB Buffer 4

44 0.4 ul - ATP @ 100mM (final concentration 1 mM)

55 0.5 ul – T4 DNA ligase (200 U)

1331 12.1 ul - H_2_0

QIAgility Protocol: “PstI-MspI_Adapters_Ligation”

To 20ul restriction digest add 5ul Adapters.

Add 15ul Ligation MasterMix and mix.

Incubate at 22°C for 2h; 65°C for 20 min; 8°C forever (program “ligate-kill-hold”)

NOTE: Completed ligation can be safely stored at -20C.

**Multiplex**

Pool 5ul from each sample ligation to a single tube (Add 20ul to empty tube prior to running robot, total volume = 500ul)

QIAgility program: “Pool 96”

Clean-up on Qiagen column (QIAquick PCR Purification Kit)

For each library do two clean-ups.

1. combine 200ul of pooled Ligation DNA and 1000ul of buffer PB in a fresh tube,

2. add 600ul to column, spin down,

3. add second 600ul to column and spin down

Follow manufacturer’s directions and re-suspend in 60ul. Combine the two clean-ups from that library.

**Amplification (25ul x 4)**

Make 4 PCR reactions for each library.

5 ul – DNA (digested library)

5 ul – 5x NEB MasterMix

2 ul – 10uM Ion Primers (F & R) @ 10uM

13 ul – H_2_0

PCR (“Ion”)

95°C (30 sec)

{95°C (30 sec), 62 (20 sec), 68°C (1 min) } 16 cycles

72°C (5 min)

8°C (forever)

For each library, pool the 4 PCR reactions and clean-up on Qiagen column (QIAquick PCR Purification Kit). Follow manufacturer’s directions and re-suspend in 50ul

Check library on 1% gel or BioRad Experion.

**ADAPTERS and PRIMERS**

| Name | oligo sequence (5’-3’) |
| --- | --- |
| ion_Y_MspI_top | CGAGATCGGAAGAGCGGGGAGCTTAAGC |
| ion_Y_MspI_bot | CCTCTCTATGGGCAGTCGGTGATCCCGCTCTTCCGATCT |
| Ion_FOR | CCATCTCATCCCTGCGTGTCTCCGACTCAG |
| Ion_REV | CCACTACGCCTCCGCTTTCCTCTCTCTATGGGCAGTCGGTGAT |
|  |  |
